# Experiences of Urban Slum-Dwelling Women With Maternal and Child Health Services During COVID-19 Pandemic: A Multi-City Qualitative Study From India

**DOI:** 10.3389/ijph.2022.1604348

**Published:** 2022-09-20

**Authors:** Krushna Chandra Sahoo, Chinki Doley, Sapna Negi, Sasmita Das, Priyanka Verma, Srikanta Kanungo, Sanghamitra Pati

**Affiliations:** Regional Medical Research Center (ICMR), Bhubaneswar, India

**Keywords:** maternal health, COVID-19, pandemic, immunization, India, child health, urban slums, SDGs

## Abstract

**Objectives:** The COVID-19 pandemic containment necessitated the diversion of substantial health care resources thus affecting the routine essential care, and posing barriers to achieving the Sustainable Development Goals (SDGs). We explored the experiences of vulnerable communities—urban-slum-dwelling women regarding maternal and child health services during COVID-19.

**Methods:** We conducted 48 in-depth interviews in four Indian states—12 in each state among urban-slum antenatal, intra-natal, and postnatal women. We used framework analysis.

**Results:** Amidst the implementation of the mandatory stay-at-home, many women acknowledged that routine immunization services and antenatal check-ups remained uninterrupted, and were mostly provided at the community level. To prevent transmission, the family members and relatives had restricted visits to the health facility during labor or post-delivery. Women preferred to have a shorter hospital stay post-delivery and reduced routine postnatal check-ups for fear of infection.

**Conclusion:** India has a variety of national and state-level programs focused on improving MCH indicators to achieve the SDGs. COVID-19 inadvertently interrupted some components of health services, insinuating the need for a disaster or pandemic-resilient MCH services delivery system.

## Introduction

The Sustainable Development Goals (SDGs 3.1 and 3.2) seek to reduce global maternal mortality rates and eliminate preventable child deaths by 2030 [1, 2]. Additionally, SDG 2.2 emphasized the importance of increasing nutritional intake among children, and pregnant and lactating women to prevent malnutrition [1]. The SDGs 3.7 and 3.8 aim to ensure universal access and coverage for maternal and child health (MCH) care—ensuring safe, quality, and affordability [1]. SDG 5 emphasized eliminating all forms of discrimination experienced by women [1]. Many MCH targets can be met through access to high-quality essential health care for the prenatal, perinatal, and postnatal periods [1, 3]. However, many low- and middle-income countries (LMICs) have considerable challenges in meeting the SDGs targets.

In India, approximately 26 million children are born each year, of which one-third die during their neonatal period [4]. Besides that, 9 out of every 45,000 women die each year as a result of a childbirth-related complication [4]. However, with timely and appropriate care, the majority of the MCH complications will be avoidable [5–7]. According to the World Health Organization (WHO), the challenge of MCH care is not limited to medical aspects; it has also been linked to neglected care during childbirth in some facilities [5]. Women are especially vulnerable during labor. As a result, effective communication and understanding among key stakeholders are required for high-quality care [5]. During the last 2 decades, India has taken numerous initiatives and prepared its healthcare system to provide universal access and quality MCH services [8, 9]. Natural disasters and emergencies, on the other hand, frequently create barriers to service provision and gradually place challenges to meeting the targets [10, 11].

The global response to the COVID-19 pandemic has unintentionally harmed community health priorities [12–16]. The COVID-19 pandemic has been largely characterized by widespread lockdowns to prevent infection spread [12–15]. The COVID-19 pandemic had a profound inadvertent effect on the healthcare delivery system and, potentially, on MCH services, particularly among the vulnerable community like urban poor—slum dwellers. The COVID-19 situation is projected to adversely affect slum dwellers compared to other urban residents, particularly in low- and middle-income nations. Prevention techniques like hand washing, self-isolation, and physical separation are not viable due to the physical, structural, and social characteristics of slum surroundings. COVID-19’s health effects in slums may be exacerbated by limited access to health care, which is common in these areas [14–16]. However, there is limited evidence regarding the health system’s readiness to provide MCH services for vulnerable communities in India during pandemics. Therefore, we explored the experiences of antenatal, intra-natal, and postnatal women receiving MCH services in India during the COVID-19 pandemic.

## Methods

### Study Design, Settings, and Participants

A phenomenological study was conducted to document women’s experiences with MCH services during the COVID-19 pandemic. We conducted this study in four Indian states: Odisha, Uttarakhand, Chhattisgarh, and Assam. Table 1 shows the key MCH indicators for these states [17, 18]. We purposefully chose one city in each state with a high population density of urban-slum dwellers. Bhubaneswar Municipal Corporation (Odisha), Rishikesh Municipal Corporation (Uttarakhand), Bhilai Municipal Corporation (Chhattisgarh), and North Lakhimpur Municipal Board (Assam) were selected for the study (Table 1).

**TABLE 1:** Key maternal and child health indicators of the selected states (Sample Registration System 2016–18) and name of the study settings (India, 2021–2022).

Study settings	Maternal mortality ratio (MMR) per 100,000 live births	Infant mortality rate (IMR) per 1000 live births	Study settings	Population (Census 2011)	Total slums population (Census 2011)
India	113	32			
Odisha	150	40	*Bhubaneswar Municipal Corporation*	843,402	163,983
Uttarakhand	99	31	Rishikesh Municipal Corporation	102,469	20,499
Chhattisgarh	173	41	*Bhilai Municipal Corporation*	625,700	214,030
Assam	215	41	North Lakhimpur Municipal Board	59,814	842

The participants in this study fell into three categories of reproductive age women: 1) those who are currently pregnant and require antenatal care, 2) those who delivered during the COVID-19 outbreak—intra-natal and post-natal women, and 3) those who had a child under the age of 1 year –immunization group. We purposefully selected 12 women from each city, four from each category. All the women are between the ages of 20 and 30—the average age of 26. All participants were representatives of a vulnerable group i.e. low income, residing in urban slums, and working in the informal sector.

### Data Collection Procedure

We conducted a total of 48 In-depth Interviews (IDIs), 12 in each state, using an IDI guide (Table 2). The IDI guide consists of open-ended questions with probes about participants’ experiences—coping strategies, and health system readiness concerning their MCH care, which includes antenatal, intra-natal, postnatal, child vaccination, and sick-infants care. We contacted approximately 57 participants with the support of community health workers (CHWs); nine declined to participate in the study due to personal issues. All participants participated voluntarily. The interviews took place between April and May 2020, during the country’s national lockdown in response to the COVID-19 first wave of outbreaks. Due to the lockdown and physical distance restrictions, the IDIs were conducted virtually, via phone calls. Apart from the interviewers and participants, no other individuals were present during the interviews. We did not conduct any repeat interviews.

**TABLE 2:** In-depth Interview guide (India, 2021–2022).

Q1. Please share your experience during the COVID-19 pandemic?
Q2. What is your experience with availing antenatal services/Intra/postnatal services/immunization services during a regular period in your community?
Probe: availability—site, day, time, staff, logistics, transport, appropriateness—technical and professional adequacy, approachability, acceptability, affordability, continuity of services
Q3. What changes have you seen in services during the COVID-19 situation?
Probe: availability, appropriateness, approachability, acceptability, affordability, continuity of services
Q4. How did you manage the care of your sick infants during the pandemic?
Q5. How did you cope with the situation?
Q6. In your view, how can we strengthen the services in an emergency?

All IDIs were held in regional languages, such as Odia, Hindi, and Assamese in this case. The IDIs were conducted by individuals who were native to the study settings and were conversant in the local language and culture. The interviewers remained resolutely involved in the community. SD facilitated IDIs in Odisha, SN facilitated IDIs in Uttarakhand, PV facilitated IDIs in Chhattisgarh, and CD facilitated IDIs in Assam. All the interviewers were female and had a nursing and clinical background, as well as training in public health and qualitative research.

### Data Management and Analysis

The IDIs were audio-recorded with the participant’s consent. Each interview lasted approximately 15–35 min. Interviews were conducted until data saturation. The IDIs were transcribed verbatim from the local language and translated into English. After transcription, the transcripts were read line by line and coded. We used framework analysis [19] and developed a code system based on the major MCH services. We coded the text segments using MAXQDA software (MAXQDA Analytics Pro 2020, VERBI GmbH Berlin). For member checking, we debriefed the findings with two participants from each state. To report the study, we followed the Consolidated Criteria for Reporting Qualitative Research (COREQ) guidelines [20].

## Results

The findings are presented under the key domains of maternal and child health services – antenatal services and nutritional care for pregnant women, intra-natal and postnatal care, immunization services, and sick infant treatment. We used quotes from participants to illustrate the findings. Table 3 shows the detailed coding tree of the results. In the description of the results, ‘majority or most or several’ of the participants refers to higher than 70 percent—in this study, more than 34 individuals viewed, “some’” refers to 20 to 70 percent, and “few” refers to usually less than 20 percent. The inter-state differences between maternal and child health services during the COVID-19 pandemic are described in Table 4.

**TABLE 3:** Detailed coding tree of the results (India, 2021–2022).

Domains	Common issues across all the study settings	Management initiatives
Antenatal services and nutritional care	• The financial crisis led to change in food consumption	• Change in timing of regular antenatal services due to diversion of frontline workers to COVID-19 duties
	• Lack of transportation services	• Provision of take-home supplementary food in advance at door-step
	• Not preferred to visit hospital due to fear of infection	
	• Reduced consultation timing	
	• Skip services because of fear of infection	
Intra-natal and postnatal services	• Early hospital discharge	• Appointment of staff in hospitals (post-graduate students)
	• The hospital required the COVID-19 testing report before admission	• Fewer restrictions to reaching the hospital
	• Limit on visitors (family members and relatives)	• Proper precaution for COVID transmission during intra-natal services
	• Prefer private hospitals which increase out-of-pocket -expenditure	• Self-arrangement for transportation
	• Reluctant for doing admission to public hospitals	
Immunization services	• Fear of child getting infected at immunization point	• Change in timing of regular immunization services due to COVID duties and reduce the spread of infection
	• Few choose a private hospital for child immunization due to fear of COVID-19	
	• Shortening the duration of the service delivery	
Sick infant treatment	• Unavailability of face-to-face medical consultation	• Encourage home-based care for children in mass media
	• Unavailability of transport services	• Facilitation of teleconsultation
	• Fear of visiting hospital	• Purchased over-the-counter medicine
	• A limited number of service providers and limited-service hours	• Stocking of essential medicines at home for emergency

**TABLE 4:** Inter-state differences between maternal and child health services during the COVID-19 pandemic (India, 2021–2022).

Domain of MCH	State-specific challenges			
	Odisha	Uttarakhand	Chhattisgarh	Assam
Antenatal services	• Limited nos of the test were allowed in pathology per day basis	• The surge in pregnant women at hospitals	• No facility for ANC checkups in PHC	• Closing of labs
	• The shutdown of private clinics	• Longer waiting time	• Rude behavior of staff	• Returned by police officials
			• Local PHC not conducting investigations	• Referral to the distant health facility
Intra/postnatal services	• No response from the ambulance	• Referral to private hospitals	• Lack of hygiene in hospitals	
	• Appointment of inexperienced staff in hospitals	• Government hospital turns COVID center		
Immunization services		• No new registration in the Anganwadi center	• Children not being weighed	• Shifted to the private hospital for the child immunization

### Category 1: Antenatal Services and Nutritional Care of Pregnant Women

Fear of infection was frequently cited as a contributing factor to accessing prenatal care during the COVID-19 pandemic. Women have voiced anxiety about antenatal check-ups at healthcare facilities because of the possibility of infection while traveling and in hospitals. Some women avoid visiting the hospital because of the travel restrictions imposed during the COVID-19-related lockdown. Many women, on the other hand, recognized that frontline health care providers had continued routine antenatal care in their communities. Few women preferred private providers, thus incurring out-of-pocket expenses.

“I used to be afraid of getting an infection when I went for a checkup. When I had stomach pain, I didn’t visit the facility for fear of infection, rather, I called the lady health worker and she advised”. (Antenatal woman, Chattisgarh)

“*I went for an* antenatal *checkup after I conceived, then corona outbreak started, so I avoided the next checkup at the routine facility and went straight to the maternity hospital to avoid chance infection*”. (Antenatal woman, Assam)

Because of physical distance norms, the providers at the facility kept a safe distance, which challenged few diagnostic services. According to the majority of respondents, to prevent the spread of infection, there was a specific time for specialists to consult at health facilities to ease the process.

“The ultrasound service required a COVID-19 test report, they did rapid Ag”. (Antenatal woman, Uttarakhand)

Despite the difficulties across all the settings, the majority of respondents said that they received the antenatal check-up while in lockdown. Several public health measures, including thermal screening at entry points, physical-distancing norms, and the mandatory use of face masks and sanitizers on hospital premises, as well as community sensitization, helped alleviate their fear of infection. The health care organization’s initiatives for seamless antenatal care included a shift in the timing of routine antenatal services at the community level due to the diversion of frontline workers to COVID-19 activities. Several participants reported that Accredited Social Health Activists (ASHAs) and Accredited Nurse Midwives (ANMs) regularly supplied Iron Folic Acid (IFA) supplements to pregnant women.

During the lockdown, however, many women acknowledged the provision of take-home nutritional supplements from Anganwadi centers in advance—Anganwadi workers supplied dry rations to antenatal women at their doorstep. Supplementary nutrition consisted of Bengal gram, groundnuts, and biscuits for pregnant and nursing mothers, and Bengal gram, groundnuts, eggs, rice chali, sweet potato, atta, soybean, rajma, Kabuli, and jaggery for children under the age of six.

“We know that pregnant women should eat a healthy diet. So, so I never skipped any meal, I used to eat the food given by Anganwadi worker in my home”. (Antenatal woman, Odisha)

### Category 2: Intra-natal and Postnatal Services

Some women prefer private clinics for intra-natal services due to fear of infection at public hospitals. At public facilities, COVID-19 test reports were required before admission, and visitors, including family members, were restricted as a precaution for COVID-19 infection. The participants acknowledge that, despite being entitled to free transportation, food, medicine, and hospitalization, under Janani Shishu Suraksha Karyakaram (JSSK) and cash benefits under the Janani Suraksha Yojana (JSY) schemes, they were not provided with these services by the public health facility. Due to their choice of private clinics, they lost their incentives and free service entitlements at public facilities while incurring out-of-pocket expenses at private facilities. Due to fear of infections, few respondents reported preferring delivering at home.

Several women reported that during COVID-19, new staff in health facilities were appointed for the continuation of the uninterrupted services. Some participants said that only one visitor was permitted in the health facility as per the COVID-19 guideline, which was implemented in all states, causing anxiety and loneliness among women. Another change mentioned by the women was their early discharge after giving birth. Few women have concerned about being discharged so soon after giving birth. However, some of them acknowledged that a shorter hospital stay had reduced the risk of infection.

“We preferred to be discharged early due to the coronavirus, and I was afraid to keep my baby in the hospital for a long period”. (Postnatal woman, Assam)

Almost all participants used some form of transport to reach the health facility. Many of them either used their vehicles or vehicles from the neighborhood. A few of them were facilitated by the police officers stationed at checkpoints to enforce movement restrictions.

Many respondents claimed that intra-natal services were prioritized over antenatal and postnatal services in health facilities. Unless there was a complication, many women perceived postnatal checkups were unnecessary given the risk of infection at the health care facility. However, some postnatal women who received services from community health workers reported that all services were provided following COVID-19 guidelines, such as maintaining physical distance, using a face mask, and sanitizing their hands.

Innovative steps like public-private partnership arrangement for transportation, fewer restrictions for intra-natal women to reach the hospital, proper precautions to prevent COVID-19 transmission during intra-natal services, and provision of adequate ambulance services only during COVID-19 were being implemented for appropriate management of intra-natal and postnatal care.

### Category 3: Immunization Services

All participants across the settings reported that getting their children vaccinated during the COVID-19 pandemic was smooth and seamless. The mothers were apprehensive of a child becoming infected at the immunization point, shortening the service delivery time, and having difficulty maintaining physical distance at immunization centers. However, due to COVID-19 duties, providers change the timing of regular immunization services. All respondents reported that immunization of children for birth doses of BCG and hepatitis B was unaffected and was given at health facilities after birth. Women who took older children for vaccination, on the other hand, reported safer vaccination protocols, such as a shorter immunization session and a prior appointment for immunization to avoid crowding and the risk of infection. A woman in Assam reported that she had to take her child to a private clinic for vaccination and pay for that.

“Earlier, on the scheduled day of vaccination, the community health workers were at the immunization point, so we went there with our children. They now call us at the appointed time. Previously, the center opened at 8 a.m.; now, they call according to the time. I was contacted by a community health worker 15 min before my name was called, and my baby was vaccinated, so I felt safe as I did not have any contact with others”. (Women having six months old infant, Uttarakhand)

### Category 4: Treatment of Sick Infants

Fear of visiting the hospital, a limited number of service providers and service hours, and the lack of face-to-face medical consultation were among the commonly perceived challenges in the care of sick infants during COVID-19. Respondents stated that they primarily sought treatment over the phone. A few participants also stated that were temporary difficulties in obtaining essential baby products like diapers and feeding bottles, as some products were out of stock, however, the local pharmacy in the neighborhood arranged.

The government encouraged and facilitated teleconsultation to continue the sick infant’s care. Aside from preventing the spread of infection, the government encouraged participants through mass media to provide home-based care for children with minor health issues. Because of the risk of coming into contact with COVID-positive patients and becoming infected, all interviewees expressed concern about taking their sick children to the hospital. As a result, the vast majority of them resorted to home-based treatment for minor ailments or purchased over-the-counter medications from local pharmacies. Many participants stated that they had some essential medicines on hand at home for emergency care.

“During the COVID-19 pandemic, I did not take my child to a hospital, but I did consult a doctor over the phone, who advised me to give liquid paracetamol”. (Postnatal women, Assam)

“Because I was afraid of infection, I preferred home remedies; when my child had stomach upset, I gave home-based oral rehydration with support from ASHA (community health worker)”. (Women having small infant, Uttarakhand)

## Discussion

To our knowledge, this is the first multicentric study to explore urban slum-dwelling women’s experiences with MCH services during the COVID-19 pandemic. Although the lockdown was strictly adhered to, many women acknowledged that routine immunization services and antenatal check-ups were not interrupted because they were provided at the community level. Certain women prefer private clinics for intra-natal care out of fear of infection at public hospitals, for which they lose their incentives and free service entitlements while incurring out-of-pocket expenditures at private facilities. Unless a complication occurred, many women believed postnatal check-ups were unnecessary, given the risk of infection in the health care facility. After all, some postnatal women who received services from community health workers reported that all services were provided in compliance with COVID-19 guidelines. Teleconsultation was encouraged and facilitated by the government to continue caring for sick infants.

Previous research from around the world has revealed a disruption of routine antenatal services during COVID-19 [21–26]. Massive media coverage instills fear of infection, which discourages them from visiting hospitals for necessary services [21, 22, 25]. Additionally, many pregnant women avoid health facilities because of public health measures – lockdown and physical-distancing, and enforcement of the stay-at-home rule [22–24]. A prospective hospital-based observational study in Jodhpur, India, found that approximately one-third of women had fewer antenatal visits than recommended, and approximately 5% had received no antenatal care [26]. The most frequently cited reasons for non-attendance at antenatal visits are transportation concerns, fear of infection, and a lack of specialists and timely care [21–26]. Many women had access to care at the community level from CHWs, despite the difficulty of providing all the necessary diagnostic services. Increased access to care prevents adverse pregnancy outcomes [16]. Hence, MCH-related emergency care should prioritize improving access to health services by strengthening community-based antenatal care.

Globally, due to the underutilization of services, the COVID-19 crisis may result in a significant increase in maternal mortality [14]. A study conducted in Jodhpur, India showed a 45 percent reduction in institutional deliveries during COVID-19 [26]. Similarly, a study in Uttar Pradesh, India revealed a 2.3 percent decline in institutional deliveries because of transportation issues and fear of infection [27]. Several studies have documented the preference for home delivery and the decline in institutional delivery [28–32]. This study paves the way for future quantitative research aimed at determining the magnitude of the problem and the severity of psychosocial aspects among vulnerable populations. The public healthcare delivery system’s response to the COVID-19 pandemic was harming both the provision and utilization of MCH services under various JSSK entitlements. This was due to movement restrictions which limit physical access, as well as limited transportation availability and perceived risk of infection at the facilities [27].

While children are less severely impacted by COVID-19 infection than adults are, non-COVID-19 related health services, particularly care for sick infants, are significantly impacted [14, 33, 34]. Our findings indicate that primary care for childhood illness and routine immunization services remained available in the community. The community health workers play a critical role in the provision of MCH care. To ensure continuous child health care, it is necessary to protect resources and actively promote children’s essential services [14, 35–37]. Data on child wellbeing at the community level is critical for responding to service delivery changes and developing future emergency care plans [37].

Digital health plays a critical role in achieving the SDGs. Many women sought virtual consultations or telemedicine for prenatal care and sick infant treatment [35, 37]. While they expressed satisfaction with the virtual consultation, some believed it influenced the amount of information disclosed to their providers. Apart from that, telemedicine is unfamiliar to many health care professionals. As a result, they have difficulty accessing virtual platforms and retrieving necessary reports [38]. It has been demonstrated that virtual home visits are effective in providing emergency MCH care. However, the most frequently cited impediment to virtual home visits is the inability to interact virtually with patients due to a lack of video, unfamiliarity with virtual software, or poor network connectivity [39].

While avoiding infection sources and limiting access to health facilities is advantageous, given the complexity of COVID-19, it is prudent to consider alternative service delivery opportunities [40]. Although, previous research has revealed barriers to MCH care for slum populations in LMICs, including in India [41]. Access to MCH healthcare services for slum families during COVID-19 was found minimal challenging. The primary reasons were the availability of community-based care, including prenatal, postnatal, and immunization services at the doorstep. For intra-natal and sick infant care, however, urban poor women experienced the same obstacles as other urban residents, notably in terms of service accessibility [42–45]. A consistent strategy for MCH care is required in the context of COVID-19, which includes strengthening the existing CHWs paradigm and integrating it with community-based organizations and referral networks. The ANM is the community health worker in the healthcare system in all four states. In Odisha, Uttrakhand, and Assam, a slum-level community volunteer known as an Urban Accredited Social Health Activist (Urban ASHA) works under the framework of NUHM. In Chhatisgarh, this community health volunteer is known as Mitanni. The role of the urban ASHA or Mitanin level slum community health volunteer in facilitating MCH services for slum women and children is crucial. In an emergency, CHWs must be recognized, trained, and supported. Our study also suggests that the role of policymakers, program administrators, and service providers from the *Ministry* of *Health and Family Welfare, National Health Mission, and* Ministry of *Women and Child Development* is critical for future emergency preparedness of essential services.

SDG-3 targets 3.1 and 3.2, commit to reducing maternal and under-five mortality rates below the global benchmark. Target 3.7 emphasized sexual and reproductive health through achieving universal access to healthcare. Target 3.8, financial risk protection, and universal access to quality essential healthcare services and vaccinations. India has an array of national and state-level programs focused on improving MCH indicators to achieve the SDGs. Among the MCH services mentioned, a web-enabled mother and child tracking system (MCTS) is being deployed to register and track every pregnant woman, neonate, baby, and kid by name for quality prenatal, intra-natal, post-natal, family planning, and immunization services. Janani Suraksha Yojana (JSY)—provides pregnant women with conditional monetary assistance for giving birth in a government health facility by providing access to competent birth attendants and emergency obstetric care. Janani Shishu Suraksha Karyakaram (JSSK) — is a free service provided by public health facilities to mothers and children.

To enhance credibility, we used to source and investigator triangulation. The information was collected from the various subgroups of women of reproductive age. Additionally, the authors came from a variety of educational and professional backgrounds, which broadened the interpretation. We used peer debriefing to avoid personal bias. Two authors independently coded and cross-checked the data to ensure consistency. We provided detailed descriptions of context, participants, and approach, which will aid in the transfer of the findings to other similar contexts. The study’s limitation was that it focused exclusively on women’s experiences; however, it did not examine the perspectives of other key MCH stakeholders, making it difficult to provide a strategic solution to the problems. Furthermore, identifying a sample that was convenient to study during a pandemic situation was the limitation of the study. The study can be replicated or adapted by other researchers in understanding different challenges faced by families from deprived population segments during the COVID-19 pandemic. Our findings may be applicable to other low-resource urban slum settings in a similar context. However, it is not applicable to other settings, including rural and tribal areas in the states.

COVID-19 disrupted MCH services, suggesting the need for a delivery system for disaster-or pandemic-resilient MCH services. Implementing global and national child health interventions in health emergencies might be difficult due to a lack of a readiness plan. Therefore, pandemic-specific modules, guidelines, and capacity-building are essential. Health-related information and community engagement can aid in emergencies. Additionally, community health workers should be compensated or reprioritized for immunization, maternal, post-natal, and nutritional needs of women and children in emergencies.

## Data Availability

The raw data supporting the conclusion of this article will be made available on reasonable request to the corresponding authors.
